# A cross-sectional study of factors influencing left ventricular myocardial work in peritoneal dialysis patients

**DOI:** 10.1016/j.heliyon.2024.e25265

**Published:** 2024-02-01

**Authors:** Xiaozhi Deng, Zhuo Huang, Junyan Yu, Yating Liu, Fang Zhu, Wenhui Zhu

**Affiliations:** a Department of Ultrasound, The Third Xiangya Hospital of Central South University, Changsha, Hunan, China; b Central South University, Changsha, Hunan, China

**Keywords:** Peritoneal dialysis, Myocardial work, Hypertension, Anemia, Hyperhomocysteinemia, Inflammation

## Abstract

**Objectives:**

To evaluate myocardial work in peritoneal dialysis patients by pressure-strain loop. To analyze the factors influencing myocardial work in peritoneal dialysis patients with preserved ejection fraction.

**Methods:**

We collected clinical data on possible effects on myocardial work in 105 maintenance peritoneal dialysis patients with preserved ejection fraction and applied ultrasonic pressure-strain loops to obtain the left ventricular global constructive work (GCW), Global work index (GWI), global work waste (GWW), and global work efficiency (GWE) of the patients. Then, the clinical data and myocardial work indices were statistically described and correlated.

**Results:**

Left ventricular hypertrophy was observed in 78 % of peritoneal dialysis patients with left ventricular ejection fraction preservation. There is a correlation between the left ventricular mass index and myocardial work indices (P < 0.05). On multiple linear regression analysis, systolic blood pressure (SBP), IL-6, and hemoglobin correlated with GCW(P < 0.05); SBP and IL-6 correlated with GWI(P < 0.05); homocysteine, SBP, TNF-α, and hemoglobin correlated with GWW(P < 0.05); homocysteine, TNF-α and hemoglobin correlated with GWE (P < 0.05).

**Conclusions:**

Using noninvasive pressure-strain loops to assess left ventricular myocardial work can provide information on cardiac function more consistent with pathophysiological changes than conventional ejection fraction. Hypertension, anemia, hyperhomocysteinemia, and inflammation influence left ventricular myocardial work in peritoneal dialysis patients, and they selectively affect one or more myocardial work indices.

## 1 Introduction

Heart Failure (HF) is a significant cause of morbidity and mortality in Peritoneal dialysis (PD) patients [1]. In addition to traditional risk factors (etiology, age, and obesity) for HF, water and electrolyte metabolism disturbances, uremic toxin accumulation, anemia, and persistent inflammatory states in the body are also risk factors for HF in chronic kidney disease (CKD) patients [2].

Measurement of left ventricular ejection fraction (LVEF) using M ultrasound or the Simpson method is the most commonly used method to assess LV systolic dysfunction. However, its sensitivity is poor and does not apply to subclinical cardiac dysfunction. Although the sensitivity of myocardial strain obtained by the speckle-tracking technique is improved, its accuracy is influenced by afterload [3,4]. Cardiac contraction is the process of energy consumption of the myocardium. Myocardial work (MW) was previously derived from pressure-volume loops measured by invasive cardiac catheters, which are limited in clinical use due to their invasive nature. The advent of noninvasive pressure-strain loops (PSL) makes it possible to evaluate MW noninvasiveness. It combines myocardial strain with noninvasive brachial arterial blood pressure and is a new method for quantitatively evaluating noninvasive left ventricular myocardial function [5]. Some studies have shown that the results agree with invasive cardiac catheterization measurements of pressure-volume loops, allowing an objective assessment of overall myocardial work [6]. PSL has good sensitivity and repeatability, can respond to changes in myocardial performance better than traditional ejection fraction and simple myocardial strain measurement, and can provide more cardiac function information [7].

The PSL technique has been used in clinical practice, including studies of MW in healthy people [8,9] and those with hypertension [10], diabetes [11], heart failure [12,13], coronary artery disease [14], and cardiomyopathy [15]. Several PSL studies in the field of chronic kidney disease have shown that myocardial function in the chronic kidney disease group is significantly reduced compared to healthy people. There were also significant differences in myocardial work among patients with different stages of CKD. PD patients are a large group of patients with end-stage renal disease (ESRD). Still, PSL has yet to be systematically studied in PD patients, let alone in-depth analysis and discussion of the influencing factors of left ventricular myocardial work in peritoneal dialysis patients, which is worth exploring. Therefore, this study aimed to evaluate myocardial function in PD patients with ejection fraction retention by applying PSL. By correlating the MW indices with the clinical data, the early detection of factors affecting the LV MW in PD patients will help guide the timely clinical adjustment of the treatment plan and reduce the occurrence of cardiovascular events.

## 2 Materials and methods

### 2.1 Subjects

This study was approved by the Ethics Committee of the Third Xiangya Hospital of Central South University (F23087). This is a cross-sectional study. Patients on maintenance peritoneal dialysis who attended the Third Xiangya Hospital of Central South University from October 2022 to April 2023 were enrolled.

Inclusion criteria: ① age ≥18 years; ② left ventricular ejection fraction >50 %; ③ glomerular filtration rate (GFR) < 15 ml/min/1.73 cm2; ④ uremia due to chronic glomerulonephritis; ⑤ maintenance of peritoneal dialysis for ≥3 months.

Exclusion criteria: ① diabetic nephropathy; ② hypertensive nephropathy; ③ lupus nephropathy; ④ aortic stenosis, left ventricular outflow tract stenosis; ⑤ congenital heart disease, cardiomyopathy, pulmonary heart disease; ⑥ After kidney transplantation; ⑦ severe arrhythmia; ⑧ poor transthoracic echocardiographic sound window; ⑨ incomplete clinical data.

### 2.2 Data collection

#### 2.2.1 Clinical data

We collected information on gender, age, BMI, length of dialysis, systolic blood pressure (SBP), and diastolic blood pressure (DBP); we also collected laboratory parameters associated with HF in patients with ESRD, including electrolytes (sodium, potassium, Chlorine, magnesium, calcium, phosphorus), hemoglobin, albumin, CRP, cytokines (IL-6, TNF-a), creatinine, urea nitrogen, parathyroid hormone, and homocysteine.

#### 2.2.2 Left ventricular morphological data and ejection fraction measurement

The ventricular septum, left ventricular diameter, and left ventricular posterior wall thickness were measured at the end of the left ventricular diastole at the left ventricular long axial section. Left ventricular mass (g) = 0.8x [1.04x (ventricular septum + left ventricular posterior wall + left ventricular diameter)3-left ventricular diameter3]+0.6, left ventricular mass index (LVMI) = left ventricular mass/body surface area. LVMI>95 (female) or >115 (male)g/m2 is the standard for left ventricular hypertrophy (LVH). Left ventricular ejection fraction (LVEF) was measured by the Simpson method on apical four-chamber and apical two-chamber view. LVEF>50 % was reserved for ejection fraction.

#### 2.2.3 Application of pressure-strain loops to obtain indices of left ventricular myocardial work

**Fig. 1:**
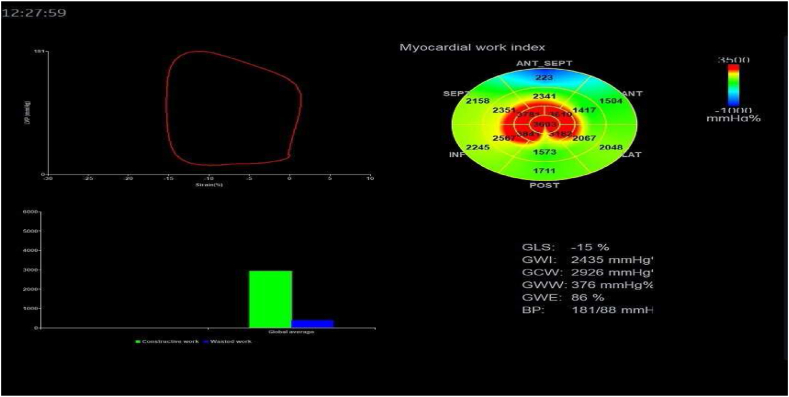
Left ventricular myocardial work analysis image of a peritoneal dialysis patient.

The ECG was connected, and the patient's apical three-chamber, four-chamber, and two-chamber images (3–5 cardiac cycles, frame rate 40–80 frames/s) were acquired using the VIVID E95 ultrasound diagnostic instrument. Export of raw images to Echo PAC workstation for image processing, determination of isovolumic systole and ejection phase by opening and closing times of mitral and aortic valves, automatic tracing of the region of interest (left ventricular endocardial border) and manual fine-tuning of unsatisfactory areas; When analyzing myocardial work, the brachial blood pressure was input, and finally obtained left ventricular myocardial indices, including global constructive work (GCW), Global work index (GWI), global work waste (GWW), and global work efficiency (GWE) [16](Fig. 1). The GWI of the LV is the sum of the work done by the left ventricular myocardium in a cardiac cycle, that is, the area of the pressure-strain loop, obtained from the integration of noninvasive blood pressure and myocardial strain pressure; GCW, or effective work, favors the LV ejection myocardium (systolic cardiomyocyte contraction and diastolic cardiomyocyte diastole); GWW is the work done by the unfavorable left ventricular ejection myocardium; GWE = GCW/(GCW + GWW)x100 % [5].

### 2.3 Statistical analysis

The statistical analyses were performed by SPSS version 26.0. The measurement data are expressed as mean ± standard deviation, and the count data are expressed as percentages. The normality of the measures was tested using the Shapiro-Wilk test. The *t*-test was used to analyze the differences between groups. Correlations between continuous variables were analyzed using the Pearson correlation coefficient. A multiple linear regression analysis was used to investigate further the independent correlation between relevant clinical information and myocardial work indices. The variance inflation factor (VIF) index was used to monitor multicollinearity, which was considered absent when VIF <10. Statistical significance was set at p < 0.05.

## 3 Results

This study initially collected data from 124 patients: 8 with reduced LVEF (<50 %), 6 with poor quality transthoracic echocardiography, and 5 with incomplete laboratory data, resulting in 105 patients with available data. The clinical data and statistical description of Ultrasonic data in this study population are shown in Table 1.

**Table 1:** Clinical data and Ultrasonic data of the study population.

Variables	Total (n = 105)
Age(y)	53.49 ± 7.42
male	62 (59 %)
BMI(kg/m^2^)	23.95 ± 2.78
systolic blood pressure (mmHg)	149.47 ± 25.11
diastolic blood pressure (mmHg)	91.78 ± 16.08
Length of dialysis performed (m)	48.07 ± 45.50
Creatinine (umol/L)	1177.58 ± 920.09
Urea nitrogen (mmol/L)	21.31 ± 8.08
Hemoglobin (g/L)	92.67 ± 21.59
Sodium (mmol/L)	140.81 ± 4.26
Potassium (mmol/L)	4.04 ± 0.76
Chlorine (mmol/L)	97.33 ± 4.93
Magnesium (mmol/L)	0.97 ± 0.14
Calcium (mmol/L)	2.15 ± 0.31
Phosphorus (mmol/L)	1.68 ± 0.52
Albumin (g/L)	28.92 ± 4.85
CRP (mg/L)	18.18 ± 20.64
IL-6 (pg/mL)	15.35 ± 18.29
TNF-a (pg/mL)	5.76 ± 5.23
PTH(ng/L)	339.34 ± 263.29
Homocysteine (umol/L)	26.16 ± 16.60
LVMI(g/m^2^)	143.07 ± 42.80
LVEF (%)	64.78 ± 8.17
GCW(mmHg%)	2089.14 ± 370.03
GWI(mmHg%)	1725.68 ± 361.03
GWW(mmHg%)	195.37 ± 63.20
GWE (%)	90.45 ± 2.29

Abbreviations: LVMI, left ventricular mass index; LVEF, left ventricular ejection fraction; PTH, parathormone; GCW, global constructive work; GWI, Global work index; GWW, global work waste; GWE, global work efficiency.

### 3.1 Left ventricular hypertrophy and myocardial work

Among 105 peritoneal dialysis patients with preserved ejection fraction, there were 82 cases (78 %) of left ventricular hypertrophy, including 38 females and 44 males. Correlation analysis showed that LVMI was positively correlated with GCW, GWI, and GWW and negatively correlated with GWE (Table 2).

**Table 2:** Correlation between LVMI and MW.

Variables	LVMI	
	coefficient	P
GCW	0.348	0.007
GWI	0.341	0.008
GWW	0.547	<0.001
GWE	−0.497	<0.001

### 3.2 Analysis of influencing factors of myocardial work

#### 3.2.1 Correlation between clinical data and GCW

Univariate analysis showed that blood pressure, age, BMI, IL-6, hemoglobin, and Potassium correlated with GCW. Multiple linear regression analysis showed SBP, IL-6, and hemoglobin were significantly associated with GCW(Table 3).

**Table 3:** Correlation between clinical data and GCW.

Variables	Univariate analysis		Multiple linear regression analysis		
	coefficient	P	standardized β coefficient	P	VIF
Age	−0.30	0.001			
BMI	0.236	0.008			
IL-6	−0.262	0.003	−0.206	0.001	1.055
systolic blood pressure	0.793	<0.001	0.747	<0.001	1.045
diastolic blood pressure	0.564	<0.001			
hemoglobin	−0.227	0.010	−0.151	0.011	1.006
Potassium	0.169	0.042			

#### 3.2.2 Correlation between clinical data and GWI

Univariate analysis showed that blood pressure, age, BMI, IL-6, hemoglobin, Potassium, and Phosphorus correlated with GWI. Multiple linear regression analysis showed SBP and IL-6 were significantly associated with GWI(Table 4).

**Table 4:** Correlation between clinical data and GWI.

Variables	Univariate analysis		Multiple linear regression analysis		
	coefficient	P	standardized β coefficient	P	VIF
Age	−0.261	0.004			
BMI	0.256	0.004			
IL-6	−0.236	0.008	−0.149	0.013	1.013
systolic blood pressure	0.791	<0.001	0.774	<0.001	1.013
Diastolic blood pressure	0.583	<0.001			
hemoglobin	−0.194	0.024			
Potassium	0.178	0.035			
Phosphorus	0.193	0.025			

#### 3.2.3 Correlation between clinical data and GWW

Univariate analysis showed that blood pressure, BMI, TNF-α, homocysteine, and hemoglobin correlated with GWW. Multiple linear regression analysis showed SBP, TNF-α, Homocysteine, and hemoglobin were significantly associated with GWI(Table 5).

**Table 5:** Correlation between clinical data and GWW.

Variables	Univariate analysis		Multiple linear regression analysis		
	coefficient	P	standardized β coefficient	P	VIF
BMI	0.279	0.002			
TNF-α	0.271	0.003	0.342	<0.001	1.088
Homocysteine	0.409	<0.001	0.544	<0.001	1.050
systolic blood pressure	0.432	<0.001	0.473	<0.001	1.043
diastolic blood pressure	0.290	0.001			
hemoglobin	−0.301	0.001	−0.227	0.001	1.091

#### 3.2.4 Correlation between clinical data and GWE

Univariate and Multiple linear regression analyses showed TNF-α (standardized β coefficient = −0.395, P < 0.001), homocysteine (standardized β coefficient = −0.645, P < 0.001), and hemoglobin (standardized β coefficient = 0.117, P = 0.015)were significantly associated with GWE.

## 4 Discussion

This study found a correlation between LVMI and MW in PD patients. When analyzing the influencing factors of MW in PD patients, it was found that MW was affected by blood pressure, hemoglobin, serum homocysteine, and pro-inflammatory cytokine levels.

Left ventricular hypertrophy is a recognized cardiovascular risk marker in patients with ESRD. This study found that most PD patients had left ventricular morphological changes under the premise of preserved LVEF. LVMI was positively correlated with GCW, GWI, and GWW, and LVMI was significantly negatively correlated with GWE as the increase of GWW exceeded the increase of GCW. This is the left ventricular remodeling of myocardia against long-term pressure load and volume load. With the intensification of myocardial remodeling, myocardial energy loss increases and work efficiency decreases, suggesting that clinical efforts should be focused on improving left ventricular remodeling and protecting myocardial function as much as possible.

### 5.1 Analysis of influencing factors of myocardial work

#### 5.1.1 Secondary hypertension

Renal hypertension is the result of a combination of multiple mechanisms. Decreased glomerular mass, impaired sodium excretion, upregulation of the renin-angiotensin-aldosterone system (RAAS), and vascular endothelial dysfunction result in peripheral vasoconstriction, extracellular volume expansion, and increased arterial stiffness, leading to increased systemic blood pressure and contributing to cardiac changes such as myocardial remodeling, tissue fibrosis, and decreased coronary reserve function [17,18]. With increased afterload, the myocardium must increase its overall contractility to maintain an adequate output per beat. However, with myocardial cell apoptosis and tissue fibrosis, myocardial pump function will gradually fail to compensate. This study found that GWI and GCW increased with blood pressure in PD patients, and hypertension was an independent predictor of GCW and GWI. It is consistent with a study that analyzed the effect of cardiovascular risk factors on myocardial function [7]. Also, GWW increased with increasing blood pressure in this study, suggesting that timely blood pressure control is essential to protect cardiac function in PD patients. In addition, no significant correlation between myocardial work efficiency and blood pressure has been found in this study, considering that most patients are still in a compensated state of myocardial pump function because the study population is PD patients with preserved LVEF.

#### 5.1.2 Anemia

Relative erythropoietin (EPO) production deficiency is associated with impaired differentiation and maturation of erythroid precursors [19]. Accumulation of uremic toxin in the circulation leads to inhibition of erythropoiesis and shortened life span [20]. Anemia in dialysis patients may be associated with iron loss. Inflammation contributes to impaired iron utilization and anemia in PD patients by modulating the expression and production of iron regulatory factors [21]. Anemia is a non-negligible factor causing LV dysfunction in CKD patients. The decrease in hemoglobin concentration in the blood, the decrease in oxygen-carrying capacity of the blood, and the body's compensatory increase in cardiac output to ensure oxygenation of vital organs. Also, due to hypoxia in cardiomyocytes, extracellular matrix synthesis increases, which can lead to myocardial fibrosis. Prolonged severe anemia can lead to total heart failure. Foley et al. showed that every ten g/L decrease in mean hemoglobin level in patients with end-stage renal (ESRD) disease was associated with LV dilatation and new and recurrent heart failure [22]. In this study, when investigating the factors affecting myocardial work in PD patients, We found that the lower the hemoglobin, the higher the left ventricular GCW and GWI, which may be a manifestation of the body to increase cardiac output. The lower the hemoglobin, the higher the GWW and the lower the GWE. It suggests that the greater the degree of anemia in PD patients, the greater the myocardial motor incoordination, the significant increase in useless work, and the tendency to decrease work efficiency.

The results of the study by Pisoni et al. indicated a 10–12 % reduction in the risk of death and hospitalization for every ten g/L increase in mean hemoglobin [23]. However, the study by Ishani et al. showed that the duration of anemia was more strongly correlated with the risk of hospitalization and mortality in patients with CKD relative to the degree of anemia [24]. Since the duration of anemia in patients could not be accurately obtained in this study, the influence of anemia duration on MW could not be analyzed. However, the duration of anemia should be more closely associated with MW.

#### 5.1.3 Homocysteine

Homocysteine (HCY) is biosynthesized from methionine in several steps and is degraded mainly through remethylation and transsulfuration [25]. Hyperhomocysteinemia (HHCY) in patients with ESRD is associated with reduced renal clearance of HCY and impaired HCY metabolism [26]. PD may facilitate HCY metabolism by removing some uremic toxins that inhibit enzymatic activity, but covalent HCY with high protein binding activity (>80 %) cannot be removed. Also, loss of folic acid in dialysis patients is a cause of HHCY. Many studies have shown that HHCY is associated with adverse cardiovascular events (such as atherosclerosis, heart attack, and heart failure, but the exact mechanisms are not fully understood [26]. Some animal studies have shown that HCY has direct adverse effects on the myocardium. HHCY increases myocardial collagen content, leading to adverse myocardial remodeling and dysfunction [27]. HHCY is closely associated with some indicators reflecting the clinical severity of the disease. For example, in the classification of chronic heart failure, NYHA classification tends to increase with progressively higher HCY levels [26]. This study also found that HCY was an influential factor in GWW and GWE; the higher the serum HCY, the more useless work the myocardium does and the less efficient it is. However, there is still clinical controversy regarding the treatment of HHCY, as some studies have shown that taking folic acid and vitamin B to lower HCY does not benefit patients and may even be harmful to the gastrointestinal tract and heart [27,28]. However, based on the strong correlation between HCY and cardiovascular disease shown in many previous studies and the present study, further clarification of its pathogenic mechanisms is necessary to improve cardiovascular prognosis.

#### 5.1.4 Inflammation and cytokines

Patients with ESRD have a microinflammatory state, and factors associated with oxidative stress, reduced cytokine clearance, and infection are responsible for the persistence of the inflammatory state [29]. In addition to the abovementioned role in anemia, cytokines can be divided into pro-inflammatory and anti-inflammatory factors according to their role in HF [30]. Pro-inflammatory cytokines induce cardiomyocyte hypertrophy, apoptosis, and fibrosis [29,31]. In this study, we collected data on ultrasensitive c-reactive protein and two pro-inflammatory cytokines (TNF-α and IL-6) and found that the majority of PD patients had elevated inflammatory mediators; correlation analysis showed that IL-6 was an influencing factor for GCW and GWI, and TNF-α was an influencing factor for GWW and GWE. Many clinical trial studies have attempted to improve heart failure prognosis by modulating inflammatory cytokine signaling pathways. However, in peritoneal dialysis patients, prevention of peritoneal dialysis-associated peritonitis by proper peritoneal dialysis practice is of utmost importance.

The pressure-strain loop is a valuable tool for early detection of cardiac dysfunction in PD patients. This study found that blood pressure, hemoglobin, serum homocysteine, and proinflammatory cytokines levels affect the myocardial work of PD patients; they have different effects on myocardial function in PD patients through specific mechanisms. Therefore, it is meaningful to comprehensively evaluate cardiac function combined with the individual state of patients when using myocardial work indices. To protect cardiac function, peritoneal dialysis patients should have adequate dialysis, control blood pressure, correct anemia, reduce HCY, and prevent infection.

#### 5.1.5 Limitations

The patients included in this study were uremia patients, most of whom had different degrees of comorbidities (such as hypertension and anemia.) and were treated with corresponding medications. However, due to the small sample size of a single center, this study could not further analyze the effects of medications patients use on myocardial function in detail.

## 6 Conclusion

In summary, this study found that the use of noninvasive PSL to assess LV MW provides information on the cardiac function that is more consistent with pathophysiological changes than conventional ejection fraction; Hypertension, anemia, HHCY, and inflammation affect LV MW in PD patients, and they selectively affect one or more MW indices.

## Data availability statement

The data will be made available from the corresponding author upon request.

## CRediT authorship contribution statement

**Xiaozhi Deng:** Conceptualization, Data curation, Formal analysis, Investigation, Methodology, Project administration, Writing – original draft. **Zhuo Huang:** Data curation, Project administration. **Junyan Yu:** Data curation, Project administration. **Yating Liu:** Conceptualization, Writing – review & editing. **Fang Zhu:** Conceptualization, Data curation, Supervision. **Wenhui Zhu:** Conceptualization, Funding acquisition, Supervision, Writing – review & editing.

## Declaration of competing interest

The authors declare that they have no known competing financial interests or personal relationships that could have appeared to influence the work reported in this paper.
